# Advanced Glycation End Products (AGEs): Biochemistry, Signaling, Analytical Methods, and Epigenetic Effects

**DOI:** 10.1155/2020/3818196

**Published:** 2020-03-18

**Authors:** Anna Perrone, Antonio Giovino, Jubina Benny, Federico Martinelli

**Affiliations:** ^1^Department of Biological, Chemical and Pharmaceutical Sciences and Technologies (STEBICEF), University of Palermo, Viale delle Scienze, Palermo 90128, Italy; ^2^Council for Agricultural Research and Economics (CREA), Research Centre for Plant Protection and Certification (CREA-DC), Bagheria, Italy; ^3^Dipartimento di Scienze Agrarie Alimentari e Forestali, University of Palermo, Viale delle Scienze, Palermo 90128, Italy; ^4^Department of Biology, University of Firenze, Sesto Fiorentino, Florence 50019, Italy

## Abstract

The advanced glycation end products (AGEs) are organic molecules formed in any living organisms with a great variety of structural and functional properties. They are considered organic markers of the glycation process. Due to their great heterogeneity, there is no specific test for their operational measurement. In this review, we have updated the most common chromatographic, colorimetric, spectroscopic, mass spectrometric, and serological methods, typically used for the determination of AGEs in biological samples. We have described their signaling and signal transduction mechanisms and cell epigenetic effects. Although mass spectrometric analysis is not widespread in the detection of AGEs at the clinical level, this technique is highly promising for the early diagnosis and therapeutics of diseases caused by AGEs. Protocols are available for high-resolution mass spectrometry of glycated proteins although they are characterized by complex machine management. Simpler procedures are available although much less precise than mass spectrometry. Among them, immunochemical tests are very common since they are able to detect AGEs in a simple and immediate way. In these years, new methodologies have been developed using an *in vivo* novel and noninvasive spectroscopic methods. These methods are based on the measurement of autofluorescence of AGEs. Another method consists of detecting AGEs in the human skin to detect chronic exposure, without the inconvenience of invasive methods. The aim of this review is to compare the different approaches of measuring AGEs at a clinical perspective due to their strict association with oxidative stress and inflammation.

## 1. Introduction

AGEs are heterogeneous molecules derived from the nonenzymatic products of reactions of glucose or other saccharide derivatives with proteins or lipids [1]. Various environmental factors, including cigarette smoke, high levels of refined and simple carbohydrate diets, hypercaloric diets, high temperature-cooked foods, and sedentary lifestyle, induce AGE production and consequently damage cell lipids and proteins [1, 2]. In this context, oxidative stress disturbs cell signal transduction, especially insulin-mediated metabolic responses, and this in turn can bring about a remarkable alteration of their normal function [3]. AGEs, through the promotion of oxidative stress, lead the activation of several stress-induced transcription factors, with the production of proinflammatory and inflammatory mediators such as cytokines and acute-phase proteins [2].

More than 20 different AGEs have been identified in human blood and tissues and in foods. In summary, AGEs can be divided into fluorescent and nonfluorescent AGEs. The most important ones include carboxymethyl-lysine (CML), carboxyethyl-lysine (CEL), pyrraline (nonfluorescent AGEs), pentosidine, and methylglyoxal-lysine dimer (MOLD) (fluorescent AGEs) [4, 5]. Although they own diverse chemical structures, their common characteristic is the presence of lysine residue in their molecule. When there is an overproduction of AGEs, an imbalance between AGEs (endogenous production and exogenous intake) and effective mechanism of the AGE detoxification system as their excretion from kidneys occurs [6]. AGE accumulation causes cumulative metabolic burden (both hyperglycemia and hyperlipidemia), inflammation, and oxidative stress [7].

In this context, oxidative stress, inflammatory response, and endothelial dysfunction are linked by binding to receptors for AGE (RAGEs) [8].

RAGEs are multiligand receptors belonging to an immunoglobulin superfamily which is expressed in a wide range of tissue, including the vasculature, lung, heart, endothelium, and neural tissue. Furthermore, they are expressed on a wide range of cells, including smooth muscle cells, monocytes, macrophages, endothelial cells, astrocytes, and microglia [9]. Under healthy conditions, RAGEs are expressed at basal levels; however, levels elevated are found under pathological conditions such as diabetes mellitus (DM), cardiovascular disease, Alzheimer's disease, cancer, and natural aging [10, 11].

The activation of RAGE induces an inflammatory cascade that starts with activation of a transcription factor nuclear factor-kappa B (NF-*κ*B) that promotes the expression of proinflammatory cytokines, growth factor, and adhesive molecules [12, 13]. Specifically, the engagement of RAGE increases oxidative stress by activating NADPH oxidase that also increases in NF-*κ*B stimulation [14]. Another group of cell surface receptor for AGEs with opposite function to RAGE, known as AGE-R1, AGE-R2, or AGE-R3, is instead involved in the regulation of endocytosis and clearance of AGEs [15, 16].

For example, AGE-R1 has been shown to be involved in pathways that decrease intracellular oxidative stress [16]. Many chronic and age-related diseases reduce the expression of AGE-R1 [16]. There is also a circulating pool of RAGE, collectively known as soluble RAGE (sRAGE), whose role remains controversial, and a minor alternatively spliced isoform of RAGE known as endogenous secretory RAGE (esRAGE) [17]. The functions of these receptors will be explained in detail and subsequently.

Due to the broad range of polarities and different structures of AGEs, there is no universally accepted method to measure them for clinical purposes. The lack of standardized methods and reference materials increases the risk to commit measurement errors and reduces the degree of accuracy and reproducibility of these methods [18]. In addition, most of the research works have been limited to monitor AGE levels in pathophysiologic conditions.

In recent years, extensive research has revealed important roles of AGEs in the progression mechanisms driving to diabetes, cardiovascular disease (CVD), hypertension, chronic inflammation, and other chronic diseases [19, 20]. Various examples of measurements of AGE's level using the qualitative and quantitative approaches have been shown with different fluid samples: high-performance liquid chromatography (HPLC), mass spectrometry (MS/MS), and gas chromatography (GC) [21]. Besides, AGEs or CML and MG derivatives are typically quantified by enzyme-linked immunosorbent assay (ELISA) [22–24]. For more than a decade, *in vivo* spectroscopy methodologies allow the measurement of autofluorescence of AGEs through detection in the human skin without the use of invasive methods [25]. Blood measurements of AGEs are indicative of their short-term presence and do not provide the state of their accumulation in tissues. Monitoring individual AGE status in healthy as well as diseased subjects may be a potent tool to slow down the onset and development of the chronic disease. This will also improve our understanding of disease pathogenesis and permit the development of new therapeutic strategies. However, methods based on the detection of autofluorescence of AGEs are limited as they exclusively measure the total fluorescent glycation. New promising methodologies for high-throughput generation of monoclonal antibodies mapped with epitopes against AGE have been applied and validated [26, 27]. Signs of progress have been obtained in identifying epitopes, reaching a high level of sensitivity and a simple analytical procedure [26, 27]. However, improvements in cultivation techniques and in the construction of specific monoclonal epitopes represent important goals to be achieved. Currently, no gold standard method is available for the detection and quantification of AGEs.

The aim of this review is to describe different approaches for AGE's measurements and provide scientific evidence of their association with oxidative stress, inflammation, and epigenetic effects.

## 2. AGE's Biochemistry

Numerous studies showed that the AGEs and the advanced lipoxidation end products (ALEs) are involved in the development and progression of chronic degenerative diseases, including diabetes [28–30], cardiovascular diseases [29, 31, 32], neurological disorder [33, 34], some types of cancer [35, 36], and all those pathologies in which the mechanisms of oxidative stress are involved, as well as the senescence processes [33].

The AGEs are heterogeneous compounds derived from nonenzymatic products of glucose reactions or other saccharide derivatives with proteins or lipids and can be formed with either exogenous or endogenous mechanisms. The ALE includes a variety of covalent molecules which are generated by the nonenzymatic reaction of reactive carbonyl species (RCS), produced by lipid peroxidation and lipid metabolism [30].

AGEs and ALEs have a similar structure because they derive from common precursors, such as CML which is synthesized by glyoxal, a product of degradation of lipid and sugar. Furthermore, they are produced by nonenzymatic mechanisms and oxidative stress that are involved in the mechanism of their formation [37].

The Maillard reaction (MR) is characterized by nonenzymatic reactions of reducing sugars with amines. The stable products of this reaction are referred to as AGEs and were initially identified in cooked foods [38]. The early stage of MR leads the formation of nonstable products, known as Schiff bases which are generated as a result of condensation reactions between the electrophilic carbonyl group of a reducing sugar with free amino groups, essentially lysine or arginine [39]. The consequent rearrangement leads to the formation of a stable ketoamine, called Amadori product. Both compounds are reversible reaction products, and they may react irreversibly with peptides or proteins to form protein cross-links. Besides, these compounds may participate in oxidation, dehydration, or polymerization reactions to give to numerous other AGEs [39, 40].

**Figure 1:**
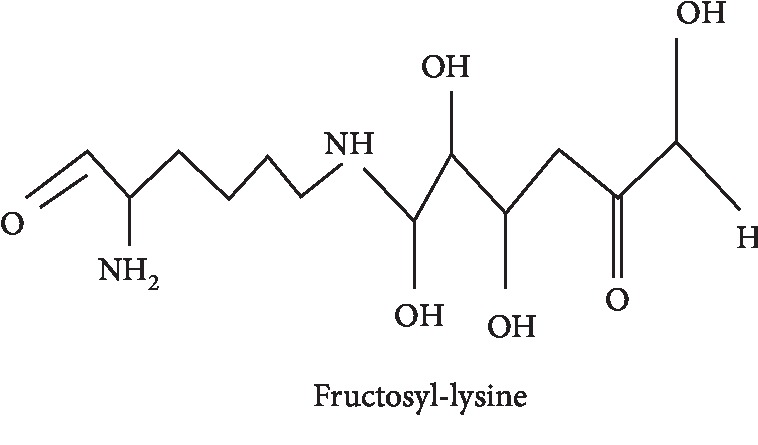
Examples of fructosyl-lysine.

In this context, reactive oxygen species (ROS) play a key role to catalyse the formation of advanced glycoxidation end products [41]. AGEs are a heterogeneous class of compounds which own diverse chemical structures. Initial investigations were concerned with the reaction between sugars and haemoglobin A1c (HbA1c), known as glycated haemoglobin in diabetes. Mechanisms of its formation include the addition of glucose molecules to amino groups located on haemoglobin *β*-chains with the formation of a Schiff base that is an unstable structure driving an Amadori rearrangement. The final product, 1-deoxy-1-fructosyl residue, owns a carbohydrate fragment attached to the HbA1c. The structure of a fructosyl-lysine molecule is shown in Figure 1. In these years, numerous other AGEs have been identified *in vivo* and *in vitro*. They are classified in different groups based on their chemical structures and ability to emit fluorescence.

These are as follows:

1. Fluorescent and cross-linked (fluorescent/cross-linked)
2. Nonfluorescent and non-cross-linked (nonfluorescent/non-cross-linked)
3. Nonfluorescent protein cross-linked
4. Fluorescent non-cross-linked

**Figure 2:**
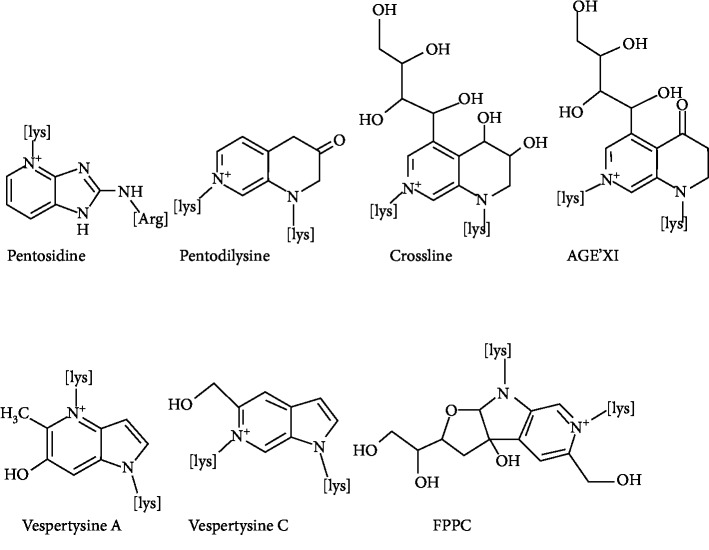
Examples of fluorescent cross-linked AGEs.

**Figure 3:**
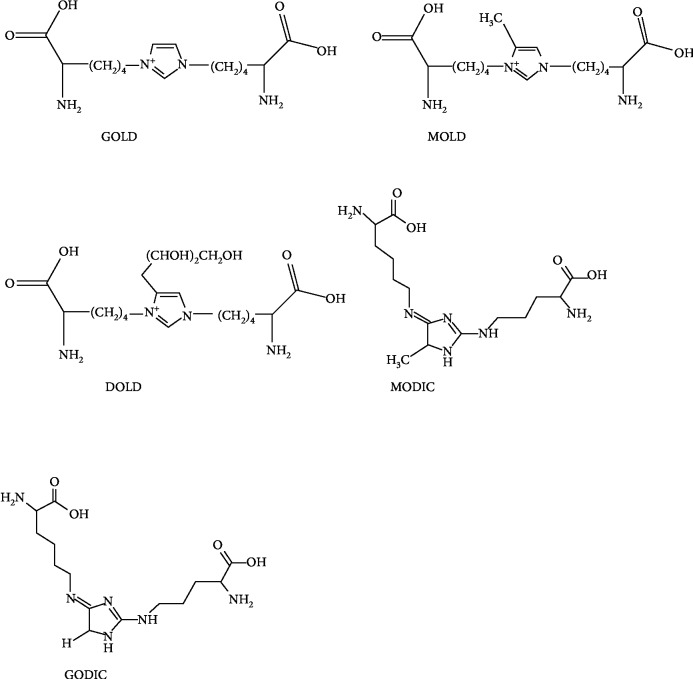
Examples of nonfluorescent cross-linked AGEs.

The first isolated and characterized fluorescent cross-linked AGEs are pentosidine (Figure 2). They are obtained from collagen and composed of an arginine and lysine residues cross-linked to a ribose but also from hexoses and ascorbic acid [42]. The level of pentosidine can be regarded as a major glycoxidative end product [43], and therefore, it is widely used as a measure of total AGE accumulation in plasma or other tissues [44]. Pentosidine is not the only natural fluorescent cross-linked AGE; other fluorescent cross-linked AGEs are pentodilysine, crossline, AGE-XI, vesperlysine A, and vesperlysine C. Several other cross-linkers but nonfluorescent compounds have been identified (Figure 3). The main AGE structures belonging to this group are imidazolium dilysine cross-links also known as glyoxal-lysine dimer (GOLD) or methylglyoxal-lysine dimer (MOLD) cross-links [45] which derive from the reaction between two lysine sidechains and two molecules of glyoxal (GO) and methylglyoxal (MG), respectively.

**Figure 4:**
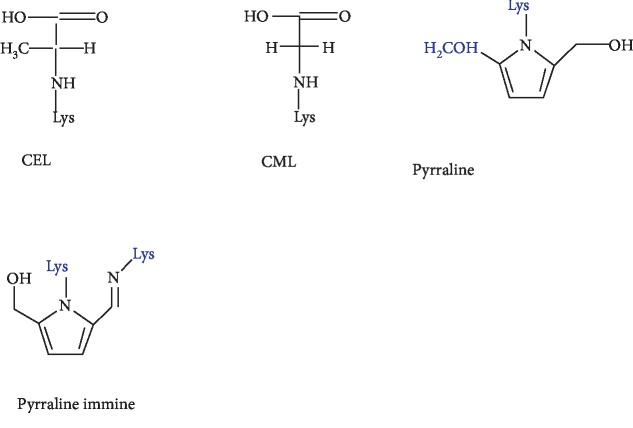
Examples of nonfluorescent non-cross-linked AGEs.

Other related cross-links of arginine, known as imidazolium cross-link derived from glyoxal and lysine-arginine (GODIC) and imidazolium cross-link derived from methylglyoxal and lysine-lysine (MODIC), have been isolated from bovine serum albumin (BSA) [46, 47]. These compounds belonging to this group are highly reactive molecules and therefore lead to the cross-linking of proteins. Important nonfluorescent and non-cross-linked AGEs include CML, carboxyethyl-lysine (CEL), pyrraline, and imidazolones [48] (Figure 4), which have been extensively studied and implicated in diabetes, inflammation, and other diseases [49–51].

These compounds are of importance *in vivo* and can be used to monitor the level of AGE accumulation during pathological conditions such as diabetes. CML represents the most prevalent AGE *in vivo* [52, 53], and it is frequently used as the AGE marker [54]. It can be formed through various pathways, including condensation of glucose with lysine of the amino group and successively rearrangement of the Amadori product, which undergoes oxidation and forms CML. Another pathway consists of the reaction directly between glyoxal (GO) and lysine of the amino group [55].

**Figure 5:**
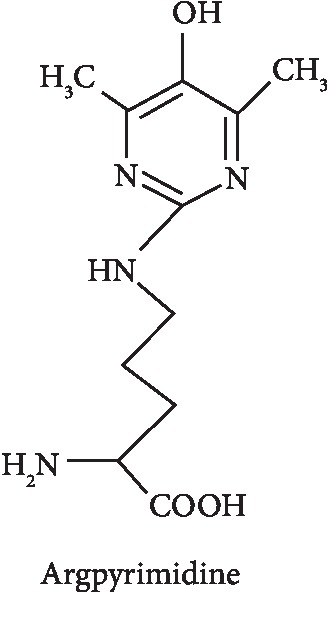
Example of fluorescent non-cross-linked AGEs.

Another important compound is pyrraline obtained by reaction between glucose and lysine residues of proteins. This compound owns importance *in vivo* and is regarded as an AGE accumulation marker throughout life and in diabetes [56]. In addition to cross-linked AGEs, a range of fluorescent non-cross-linked AGEs are detected in the blood of diabetic patients (Figure 5). They are structurally like fluorescent cross-linked AGEs, except that one of the bonds links the heterocyclic part with the amino acid and is replaced by the N-H bond.

They also involve promoting a variety of undesired changes at cellular and tissue levels through the mechanism of biological receptor [57].

**Figure 6:**
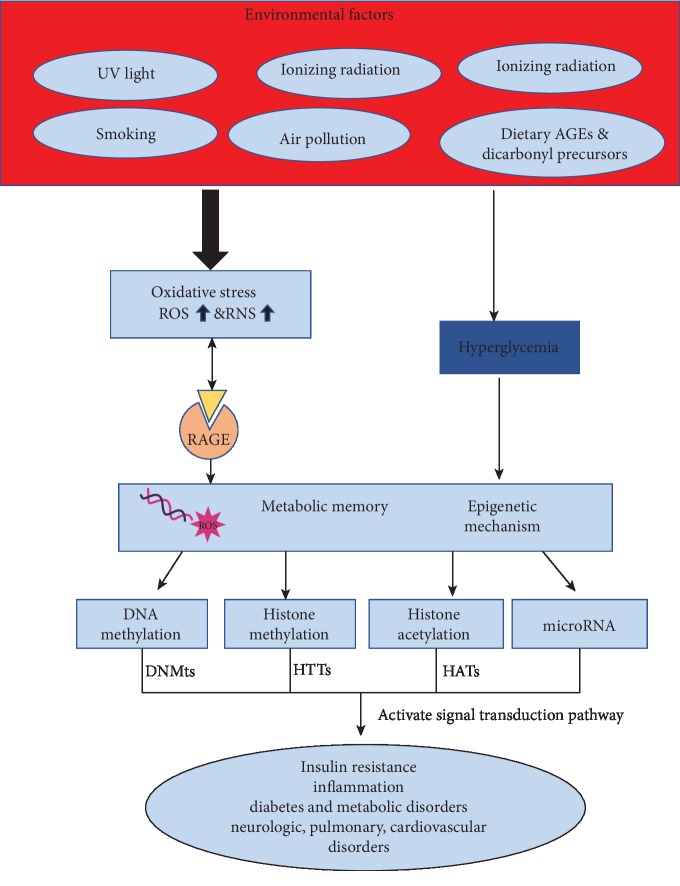
Schematic representation of AGEs' formation and their biological effects.

Argpyrimidine is a typical example of AGE structure belonging to this family of fluorescent non-cross-linked AGEs. It is formed from arginine and methylglyoxal through the Maillard reaction, and it has been studied for its food chemistry purposes and its potential involvement in elevated oxidative stress content in several tissues [58, 59]. AGEs may derive from an exogenous source, through food consumption and tobacco smoke (Figure 6). During the normal metabolic processes of a healthy organism, AGEs and their precursors are also formed at lower rates, while they are increased in diabetes [54], atherosclerosis [60], and other chronic pathologies. Besides, various environmental factors, such as Western diet, cigarette smoke, or inflammation, induce a high AGE production. For example, cigarette smoke contains high concentrations of GO and MG and provokes the accumulation of other AGEs in plasma of smokers [61].

Glyceraldehyde, in the form of glyceraldehyde 3-phosphate, an important intermediate in the metabolism of glycolysis, is involved in the formation of GLAP compound (glyceraldehyde-derived pyridinium), a compound glyceraldehyde-derived advanced glycation end product (AGE). Specifically, when glyoxidation occurs, new compounds are formed, like methylglyoxal (MG) and glyoxal. These in turn can also react with proteins. In this case, MG reacts mainly with arginine while glyoxal reacts with lysine residual. Advanced glycation derived from glyceraldehyde (GLAP) is elevated in oxidation stress, inflammatory, and/or diabetic conditions, endothelial dysfunction, and vascular inflammation [62–64]. High levels of GLAP have been detected in Alzheimer's disease [65]. GLAP, such as pentosidine and other AGE compounds, is a biomarker involved in metabolic diseases such as diabetic and vascular complications [66, 67]. A recent prospective study in European populations has shown that GLAP may promote colorectal cancers (CRC) through proinflammatory and prooxidative ways [68]. However, although circulating GLAP were not associated with the overall risk of CRC, a positive association was found. Measurement errors of interpretation may be underestimated. The detection of GLAP was conducted through ELISA. Due to the underestimation of the circulating GLAP detection, further research is needed to clarify the role of toxic carbohydrate metabolism products in the pathogenesis of CRC development. Once again, more precise and large-scale techniques are needed.

Moreover, variations of circulating AGEs may also be influenced by the genetic ability to detoxification mechanisms against the accumulation of AGEs [69]; therefore, the content of AGEs in the organism depends not only by the rate of their formation but also by the ability to being removed through intrinsic detoxifying pathways. Several possible mechanisms of detoxification against AGEs include reduced glutathione (GSH) which catalyzes the conversion of GO and methylglyoxal (MG) to the less toxic D-lactate [70]. Other enzymatic systems include fructosamine kinases [71], which act on phosphorylating and destabilizing Amadori products leading to their spontaneous breakdown.

## 3. RAGE: Receptor for AGEs

**Figure 7:**
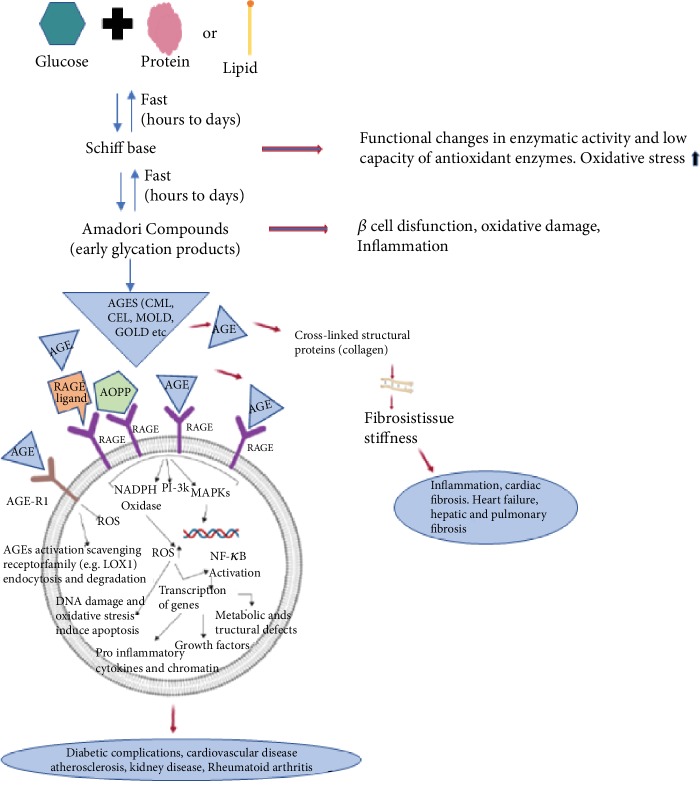
Biochemical formation of AGEs, their signaling, and molecular signal transduction that lead to pathological effects.

The RAGE is a transmembrane protein belonging to the immunoglobulin superfamily of cell surface receptors, encoded by a gene on chromosome 6 near the major histocompatibility complex III [72]. The gene consists of 11 exons, and typical variations of this gene have been described [73]. RAGE consists of an extracellular region characterized by the immunoglobulin domains of types V1, C1, and C2, transmembrane-spanning domain, and short cytosolic tail [9, 11]. The extracellular part of a RAGE molecule is composed of a variable (V-type) domain, which is followed by two constant (C-type) domains and represents the main binding sites for various ligands, while the cytosolic tail of RAGE is essential for signaling [9]. Domains of type V1 and C1 of RAGE bind a large variety of molecules (Figure 7), not only AGEs (endogenous or food-derived) but also advanced oxidation protein products (AOPPs), involved in oxidative stress [35], *β*-amyloid related to Alzheimer's disease [10, 74], calcium-binding S100 proteins linked to several human cancers [75], and high-mobility group box-1 (HMGB) expressed in cancer and inflammation [35, 76]. RAGE receptors have been identified in a series of organs and tissues, but their highest concentration is in the lung, heart, and skeletal muscles. Furthermore, they are expressed on a wide range of cells including smooth muscle cells, monocytes, macrophages, endothelial cells, astrocytes, and microglia [8]. Under normal and healthy conditions, RAGE is expressed at basal levels; however, levels elevated are found under pathological conditions or chronic inflammation such as diabetes mellitus (DM), cardiovascular disease, Alzheimer's disease, cancer, and natural aging [10, 11]. Furthermore, the activation of RAGEs by AGEs or other ligands also transduces multiple signals, such as the mitogen-activated protein kinases (MAPKs), extracellular signal-regulated kinases 1 and 2 (ERK), p21^ras^, p38, and Janus kinase [77]. The binding of ligands to RAGE activates various signaling pathways which induce activation of the transcription factor nuclear factor-kappa B (NF-*κ*B) that increases the transcription of many proinflammatory genes [10, 78], MAPK, transducers, and activators of the Janus kinase signal from transcription (JAK-STAT), and phosphoinositol 3 kinase, and consequently causes inflammatory, proliferative, angiogenic, fibrotic, thrombogenic, and apoptotic reactions [79, 80]. The binding AGE/RAGE increases the levels of ROS [81] through activation of NADPH oxidase and mitochondrial pathways [14]. Consequently, the activity of superoxide dismutase (SOD), catalase and indirectly other endogenous antioxidant defense is decreased, such as glutathione (GSH) and ascorbic acid [82]. In fact, oxidative stress is highly related to glycation, since the depletion of GSH also reduces the activity of Glyoxalase-I (Glo-1), thus increasing concentrations of glyoxal and methylglyoxal (MG), formed nonenzymatically as products, mainly in glycolysis, and leads to the formation of AGEs [83]. It is interesting to note that AGE-R1 is downregulated by high levels of AGE [84]. Furthermore, other studies have shown that AGEs increase the oxidation of LDL and promote the development of atherosclerosis [85, 86]. Glycated LDLs are therefore more sensitive to oxidation [86]; they are reduced with difficulty and promote the formation of antibodies that bind the AGEs located in the vessel wall, which amplify the development of vascular inflammation and atherosclerosis [87]. Glo-1 overexpression has beneficial vascular effects, reduces ROS, and protects against atherogenic LDL formation [63, 88].

In patients with type 2 diabetes mellitus, circulating AGE levels are positively correlated with RAGE mRNA expression and oxidative markers, such as protein carbonyl, advanced oxidation protein product (AOPP) generation, and lipid peroxidation [89]. In this context, RAGE is a strong upregulation of NF-*κ*B that leads to transcriptional activation of different genes involved in inflammation including cell adhesion molecules like E-selectin, ICAM-1, and VCAM, and proinflammatory cytokines (IL-6, 8; TNF-*α*) [90, 91]. The chronic inflammation signals, oxidative stress, and high AGE content carry out the process of tumor initiation, allowing the constitution of a microenvironment that initiates a premalignant niche [92]. All these evidences always happen through the link AGEs or ligands and RAGE. The AGE-RAGE bond promotes the downstream enhancement of the enzyme NADPH oxidase 2 (NOX2), consequently ROS production, and NF-*κ*B activation, which in turn promotes further RAGE expression. Signaling axis AGE/RAGE/NOX2/NF-*κ*B promotes inflammation and cancer [93]. All four components of this axis are expressed in high levels in tumor tissues compared to the control [94]. Reduction or inhibition of NF-*κ*B activation is the goal of many therapeutic interventions for diabetic, cardiac, pulmonary, and all other pathologies dependent on AGE-RAGE interaction. For example, blockade of the AGE/RAGE pathway is thus a potential target to control or slow down atherosclerosis progression [95]. Another group of cell surface receptor for AGEs with opposite function to RAGE, known as AGE-R1, AGE-R2, or AGE-R3, is instead involved in the regulation of endocytosis and degradation of AGEs [16, 88]. AGE detoxification is mediated by other receptors including scavenger receptors class A, type II (MSR-AII), and class B, type I (MSR-BI, CD36) [15, 96].

As previously said, the AGE linked with specific RAGE has been involved in the activation of critical signaling pathways that are responsible for activating the genes linked to the inflammatory responses [97]. Several AGE accumulation effects cause stress in endoplasmic reticulum (ER) and induce apoptosis or activate NF-*κ*B via signaling cascade. ER stress, for example, is coupled with the activation of MAP kinases [98]. Genes involved in inflammation are activated by certain transcription factors, including NF-*κ*B, which has been phosphorylated by members of the MAPK family [99–107]. Another mechanism involved in the regulation of protein synthesis during ER stress concerns the phosphorylation of the subunit of eukaryotic initiation factor 2 (eIF2) which, in turn, involves the activation of NF-*κ*B [108] and many cellular genes that are largely antiapoptotic under ER stress [109]. Apoptosis is a form of programmed cell death or “cellular suicide.” It is a natural cell death, and it is different from necrosis, in which cells die due to injury. It plays an important role in wound repair by preventing a prolonged inflammatory response and excessive scar formation. An abnormal functioning of the complex apoptotic mechanism causes slow wound healing and a high state of inflammation. Diabetic subjects, having high AGE content, are characterized by slow wound healing and high states of inflammation [110, 111]. It is clear that ER stress activates multiple signal transduction pathways including eIF2*α*, MAPK, and NF-*κ*B [99, 111, 112]. These pathways may be partly shared or converge on common downstream effectors [99, 112]. For example, AGEs induce ER stress and stimulate the expression of Cox2 through the eIF2*α*, p38 MAPK, and NF-*κ*B pathways in human chondrocytes [113]. These results explain that cartilage degradation in osteoarthritis is associated with latent ER stress [113]. The accumulation of AGEs in the tissues is detrimental to the survival of cells that make it up. The presence of reactive oxygen species induced by AGEs (ROS) raises carboxylic content of cellular proteins leading to apoptotic events. However, these mechanisms are not always clarified. Autophagy is significantly related to apoptosis [114]. Apoptosis and autophagy were related to reactive oxygen species (ROS) production, and in this context, AGEs induced autophagy [113].

A type of RAGE that involved the pathway of intracellular oxidative stress is AGE-R1 that has been shown to be involved in pathways that decrease intracellular oxidative stress [16]. These receptors are present on the surface of different cell types, including macrophages, vascular smooth muscle cells, lung cells, and endothelial cells, and activate scavenging receptor mechanisms [66, 70, 71].

There is also a circulating pool of RAGE, collectively known as soluble RAGE (sRAGE), whose role remains controversial, and a minor, alternatively spliced isoform of RAGE known as endogenous secretory RAGE (esRAGE) [17, 114, 115]. These receptors have been detected in human plasma. sRAGE is generated by proteolytic cleavage of native membrane receptors mediated by matrix metalloproteinases (MMP) [17, 114]. It has been found in plasma and owns a ligand-binding domain but lacks the transmembrane and cytoplasmatic domains [17, 115]. In animal studies, administration of sRAGE prevented and slowed atherosclerosis [116, 117]. In another study, administration of sRAGE improved retinal neuronal dysfunction in diabetic mice [118]. These results [115–118] suggest that sRAGE acts by trapping, binding, and eliminating circulating AGEs. Another study, instead, shows that sRAGE represents a marker of RAGE expression of tissues and disease activity [119]. For this reason, the exact pathophysiological role of these soluble variants remains controversial and must be further investigated by future studies. Frequently, esRAGE and sRAGE are measured using the enzyme-linked immunosorbent assay (ELISA). The sRAGE measurements include esRAGE in order to evaluate the relationship between sRAGE or esRAGE and inflammation. Human studies have reported a reduction in serum of sRAGE levels in subjects with inflammatory conditions such as atherosclerosis, obesity, rheumatoid arthritis, and chronic obstructive pulmonary disease [120, 121]. In contrast, other studies on diabetic patients have shown that sRAGE is positively correlated with circulating inflammatory markers such as TNF-*α* and MCP [122]. The opposite results of the sRAGE content between patients with diabetes and people with other inflammatory conditions are demonstrated by increased levels of matrix metalloproteinases, since high circulating AGEs, frequently in diabetes, are linked with increased production and expression of metalloproteinases of matrix, thus increasing the proteolytic cleavage of sRAGE expressed by the cell surface [123]. Serum sRAGE levels have been shown to be five times higher in healthy subjects [124]. Extensive studies on the polymorphisms of the RAGE gene will be able to clarify the roles of sRAGE and esRAGE at the cellular level.

## 4. Measurements of AGEs

The complex nature of glycation and the widest range of chemical structures and physical properties lead to difficulties in their measurement. The goal is the detection of all AGE compounds in a single run. No standardized method exists, and there is no possibility to compare AGE results in a meaningful way between different laboratories [18]. In this context, it is difficult to draw any firm conclusion about the relative toxicity of these wide classes of AGEs.Table 1 summarizes the different methodologies for the analysis of AGE with their advantages and disadvantages.

Methods for identification and quantification of AGEs in biological samples include instrumental methods and immunochemical methods. The instrumental methods include the following: (1) spectrofluorimeter [125], (2) high-performance liquid chromatography coupled with mass spectrometry (HPLC/MS) [126], (3) gas chromatography coupled with mass spectrometry (GC-MS) [127], (4) liquid chromatography coupled with tandem mass spectrometry (LC-MS/MS) [128], (5) HPLC with fluorescent detection [129], and (6) method based on ultra-high-pressure liquid chromatography (UHPLC) [130]. Immunochemical methods are mainly enzyme-linked immunosorbent assay (ELISA) [131, 132] and Western blotting [133], using antibodies specific for certain AGE structures.

Estimation of serum, urine, and saliva AGEs might be measured by spectroscopic and fluorimetric methods. However, the amount of AGEs quantified by this method provides only the quantification of fluorescent AGEs. The determination of fluorescent AGEs is quite simple. Briefly, samples are diluted (50-fold for serum, 10-fold for saliva, and from 10- to 200-fold for urine) with phosphate-buffered saline (PBS) at pH 7.4 and the amount of AGEs is measured at *λ*_ex_ = 370 nm and *λ*_em_ = 440 [10]. The specific fluorescence of AGEs is expressed in arbitrary units.

A simple, noninvasive, and rapid *in vivo* technique allows the detection of AGEs in the skin, by use of autofluorescence spectroscopy [25].

This method employs an ultraviolet A radiation of 370 nm (at low intensity) interacting with AGEs in the skin. The light emission is measured at 440 nm from the skin using a spectrometer portable.

Importantly, many studies have shown the validity of AGE reader in the skin and its significant correlation to total AGE content measured by high-performance liquid chromatography (HPLC), which however requires invasive collection of skin samples [25, 60, 134]. In particular, authors have shown that skin AGE levels of different patient groups and healthy controls, measured by AGE reader, are significantly correlated to levels of both fluorescent (e.g., pentosidine) and nonfluorescent AGEs (e.g., carboxymethyl-lysine and carboxyethyl-lysine) assessed in skin biopsies [25, 60, 135], even if the major contribution in fluorescence comes from fluorescent AGEs. Moreover, skin AF has been shown to be highly a significant predictor of long-term diabetic complications (5-10 years) compared to short-term glycemic memory reflected from haemoglobin A1c (HbA1c) (3-6 months) [136, 137] because skin AF represents the “long-term memory” of cumulative metabolic stress compared to HbA1c or other conventional risk factors (e.g., smoking and blood pressure).

It has been observed that skin AF is also correlated with the progression of complications of renal failure and cardiovascular ones, being a marker to produce advanced glycation end products [60, 127].

Different studies describe the influence of different absorptions of excitation or emission of light by darker skin colours which result in lower values than in subjects with fair skin colours [138]. Recently, a new algorithm based on comparison on these results has been validated to assess skin AGEs independently of skin colour [127]. Spectroscopic autofluorescence (SAF) is considered a noninvasive, rapid, accurate, and safe assessment of AGEs in the skin [139] and is associated with oxidative stress, in which this latter induces complications of diabetes, including stroke, neuropathy, retinopathy, and nephropathy [140]. This technique is easy to use and is applicable to epidemiological studies with healthy subjects and those having risk of developing the disease, even considering any limiting factors, explained later.

Although SAF plays an important role in characterizing and predicting an individual's metabolic health, autofluorescence measurement methods have several limitations that must be improved to increase their relevance in the clinical setting. SAF measurement may not represent only the AGEs in the skin content. The presence of endogenous fluorescent signals from cutaneous fluorophores (i.e., nicotinamide adenine dinucleotide) has the same excitation and emission ranges (respectively, 350–410 and 420–600 nm) and may interfere with the correct total fluorescence measurement [135]. SAF method is currently used for research purposes, and future use in the daily clinic would require further studies. Its advantages constitute one positive perspective for future patient monitoring in order to limit the incidence of diabetes complications and various chronic diseases in which oxidative stress and inflammation are involved. So far, SAF measures contain various technical aspects of problems that are still being improved. Therefore, further research studies will aim to improve and consolidate this technique. A research group has developed a new imaging system with transmission geometry for SAF measurement in order to improve possible and future diagnostic performance [131]. However, these measurements were carried out on a limited number of Korean subjects [131]. Soon, it is hoped that SAF will be available as a noninvasive technique, relatively cheap and easy to use in ambulatory practice.

Sophisticated and expensive laboratory techniques, such as mass spectroscopy and gas and/or liquid chromatography, provide exceptional sensitivity and specificity for detection and quantification of specific AGEs, but there is a need for highly qualified personnel and high costs, and for these reasons, these techniques have difficulties in widespread use. For example, liquid chromatography coupled with mass spectrometry gives higher sensitivity than the fluorescence detection method (HPLC with fluorescence detector) [127]. Liquid chromatography coupled with tandem mass spectrometry (LC-MS/MS) has been used for the analysis of nonvolatile compounds [121]. One major advantage is that usually no derivatization step is required. Besides, the use of a tandem MS system offers improved sensitivity.

Gas chromatography coupled with mass spectrometry (GC-MS) has been widely used for the analysis of CML and CEL in urine and protein hydrolysates [134] or used to quantify the amount of CML in different food samples [119]. A new method based on ultra-high-pressure liquid chromatography (UHPLC) is involved in the use of smaller particles (1.7 *μ*m) which allow faster analysis and better resolution.

In general, measurements of N-carboxymethyl-lysine, pentosidine, and methylglyoxal (MG) which are used as AGE biomarkers have mainly been analyzed by LC-MS/MS and competitive ELISA [124, 125]. This method uses monoclonal antibody specific for AGE, developed to measure of AGEs in different human tissues, biological samples, and foods. This procedure is relatively simple, fast, and inexpensive, and there is no need for sophisticated laboratory equipment. However, the disadvantages are as follows: (1) the lack of enough antibody specificity, depending on the commercial kit used, and (2) interference with glycation-free adducts [136, 137]. In this context, the assessment of AGE by immunoassay does not measure AGE levels in absolute concentrations but rather in arbitrary units with or without normalization to a reference AGE glycated protein standard [22, 125]. However, some problems result from the use of biological samples rather than food samples.

In recent years, new promising methodologies for high-throughput generation of monoclonal antibodies mapped with epitopes against AGE have been applied and validated. For example, recent studies have allowed the generation of new monoclonal antibodies destined for carboxymethyl-lysine [26, 27]. Monoclonal antibodies have the potential to detect and quantify single glycated epitopes, but the effective and specific ones currently available are limited. Other available monoclonal antibodies have not described epitopes and do not show specific bonds [137]. Therefore, there is a great need for monoclonal antibodies with a well-defined binding pattern, to bridge the specificity gap and create a basis for better analytical capacity.

Immunological methods could potentially offer several advantages in identifying these compounds, such as the rapid achievement of results, greater detection sensitivity, and simpler application [135, 139, 140]. However, improvements in cultivation techniques and in the construction of specific monoclonal epitopes represent important goals to be achieved. Currently, no gold standard method is available for the detection and quantification of AGEs. A possible explanation for this is that there is no internationally recognized standard unit of measurement for expressing AGE levels, unlike other measurable molecules. These molecules are characterized by complex structural and molecular heterogeneity. As a result, it is extremely difficult to compare the results between different laboratories.

ELISA have been used extensively for the detection of AGEs in serum or other biological samples or food matrices. This technique exploits the use of monoclonal or polyclonal antibodies [139, 140]. It has yet some limitations represented by (1) lack of antibody specificity, (2) high background responses due to the significant content of protein glycation adduct [141], and (3) interference with glycation-free adducts [142] due to pretreatment techniques, such as heating and alkaline treatment [22, 143]. Major improvements have been recently made, and the use of ELISA has made important contributions to be easily used in the measurement of CML in plasma and urine [144] and in various foods [145]. A significant relationship between CML determined by ELISA and HPLC-ESI-ITMS/MS analysis was demonstrated recently. This involves the implementation of ELISA in food CML/AGE screening [145].

Serum AGEs, RAGEs, and sRAGEs may be measured by Western blotting. Samples are initially separated by SDS-PAGE (10% gel), and gel is electrotransferred to polyvinylidene difluoride membrane. Nonspecific binding sites of proteins are blocked with TBST (Tris-buffered saline containing Tween 20) with 10% powdered skimmed milk. The blocked membrane is incubated at room temperature for 1 hour with rabbit anti-human AGE polyclonal antibody, goat anti-rabbit IgG, or polyclonal goat Ab against human RAGE. Bands are visualized and analyzed [36] by an enhanced chemiluminescence advanced detection system and then exposed to X-ray film (e.g., Kodak).

Western blotting (WB) is an analytical method for identification and quantification of a specific protein such as AGE-modified ones in biological samples. Fluorescent-labeled primary enzymes or antibodies with ability to bind to the specific antigen are generally employed in Western blotting. Although WB is accepted as a routine protein analysis technique, it has benefits and limitations [145, 146]. WB has a high sensitivity. Due to its ability to detect up to 0.1 nanograms of protein in a sample, the technique could prove to be an effective early diagnostic tool. In addition, the WB technique owes its specificity to two major contributing factors. The gel electrophoresis step order proteins of different size, charge, and conformation, representing fundamental clues on the size of the protein or polypeptide of interest. Subsequently, the specificity of the antibody-antigen interaction acts as a second major factor for a specific detection. All these aspects allow to selectively detect a target protein even in a mixture consisting of thousands of different proteins. Despite its high sensitivity and specificity, WB could still produce incorrect results [145, 146]. For example, false positives could occur when an antibody reacts with an unintended protein. A false negative, on the other hand, could occur if large proteins do not have enough time to properly transfer to the membrane. For this reason, distorted, faded, or even multiple bands could appear providing wrong misunderstood results. WB requires high costs due to the use of antibodies, expert analysts, and laboratory equipment. The technique requires precision in each phase, and a trivial error in one step could compromise the whole process. Finally, the equipment needed for detection and imaging (chemiluminescent, fluorescent, radioactive, or laser detection systems) can be too expensive [145, 146]. Western blotting has the benefit of allowing simultaneous detection of several targets, whereas ELISA can detect only one product. It is also possible to determine the size of the target to be analyzed, and therefore, it is also possible to (semi-) quantify the AGE or RAGE of interest by running a gel with the sample containing the target protein of interest and in parallel with a quantity of standard. Compared to ELISA, Western blotting is more time-consuming and has a higher demand in terms of experience and required experimental conditions (i.e., protein isolation, buffers, type of separation, and gel concentration).

In conclusion, the most suitable technique for specifying the application depends on several available factors such as trained personnel, available laboratory, financial resources, and object of the study (AGE concentration, number of samples, type of samples, etc.). However, the chosen methodology is important to provide reliable data, especially if these data are a reliable tool to monitor the healthy subjects, the effects of therapeutics, and the progression of a pathological status.

## 5. Epigenetic Effects of AGEs

AGEs have negative effects on cells and tissues, and the enhanced accumulation in hyperglycemic patients has caused atheroarteriosclerosis and type II diabetes. Evidence has shown that epigenetic mechanisms are induced by AGEs due to the RAGE. These processes can be reduced by ROS scavengers such as oligo- and polysaccharides and L-carnosine, catalase, and rhamnose-rich molecules [147]. Cytotoxicity associated with hyperglycemia can occur not only in the skin in other tissues due to the cross-linking of collagen with Maillard reaction products with induced cytotoxic effects on cells. Epigenetic processes are those mechanisms that induce variation in gene expression and phenotype due to genetic modifications that do not deal with alterations of the DNA sequence. Epigenetic changes can be divided into their short- or long-term effects. The short-term effect is a rapid response to an environmental factor that is typically not transmitted in the filial generation. The long-term effects induce a persistent modification that remains as “memory,” and it is heritable, typically in response to a long-term and intensive environmental stimulus [148]. It is worthy to notice that also a transient modification to a short environmental change may provoke a permanent epigenetic effect [138].

The epigenetic effects of AGEs have been investigated in relation to the occurrence of diabetes and other chronic diseases. Together with tumor necrosis factor, AGEs induced MMP-9 promotor demethylation although this complex mechanism is still unclear. DNA methylation of MMP-9 promoter is mediated by the involvement of GADD45a, growth arrest, and DNA damage protein 45 that is a member of a small family of stress-responsive genes [144]. GADD45a was linked to diabetes and diabetic cardiomyopathy, and it was shown to interact with proteins involved in DNA methylation. The real role played by GADD45a in MMP-9 promoter demethylation has been partially recently elucidated. GADD45a expression was induced in the skin of patients showing diabetic foot ulcers. In addition, this gene was upregulated in diabetic rats, and in humans, keratinocyte (HaCaT) cells were treated with AGEs [147]. A partial positive correlation between the expression of GADD45a and MMP-9 was observed. The knockdown of GADD45a supported the upregulation of MMP-9 transcription through the downregulation of demethylation in the MMP-9 promoter [148].

Recent studies have highlighted the role played by epigenetic mechanisms in the well-studied phenomenon of metabolic memory of diabetes [149]. The pathological effects of hyperglycemia are due to four basic mechanisms: PKC activation, enhanced production of AGEs, enhanced glucose flux due to polyol pathway, and upregulation of hexosamine pathway activity [150, 151]. Hyperglycemic status has been associated with the activation of histone modifications that provoke the enhanced expression of inflammatory genes [152]. Interestingly it was observed that these chromatin remodeling changes, as well as histone modifications in promoters, were maintained even if glucose levels become normal implying that epigenetic changes are closely associated with metabolic memory. A work conducted in human podocytes has shown that AGEs downregulated NAD-dependent deacetylase sirtuin 1 (SIRT1), provoking enhancement of acetylation of key transcription factors such as STAT3, NF-kappaB-p65 (NF-*κ*B (p65)), and FOXO4 [153]. The acetylation of STAT3 upregulated proinflammatory genes causing podocyte apoptosis and consequently kidney disease. Further studies are needed to investigate genes targeted by epigenetic mechanisms of dietary AGEs. Epigenetic mechanisms are the base of the regulation of hyperglycemic memory that is the phenomenon that drives a hyperglycemic state in the absence of causing factors but associated with a previous hyperglycemic state (Figure 6). This pathological status has been associated with oxidative stresses and occurrence of AGE as well as the induction of pathways of mitogen-activated protein kinase [154, 155]. The induction of ROS in mitochondrial structures has been associated with the induction of pathways of hyperglycemic disease such as the upregulation of polyol pathways, induction of protein kinase C signaling, and enhanced production of AGEs [156]. The epigenetic mechanism has been linked to the expression of NF-*κ*B-p65 [157] and the occurrence of permanent epigenetic marks such as enhanced levels of H3K4 and reduced H3K9 methylations at the promoter of p65 gene [158]. The methylation patterns are caused by the activity of enzymes with opposite effects such as histone methyltransferase SET7 and histone demethylase LSD1. Silencing of SET7 has been associated with metabolic memory through the action of DNMT1 methylation that directs it to its degradation via proteasome [159]. On the contrary, LSD1 seems to avoid DNMT1 degradation eliminating the methylation mark. Indeed, metabolic memory should be caused by the competition between the epigenetic action of SET7 and LSD for methylation changes at DNMT1. A recent cohort study has shown the link between specific histone modifications and glycemic memory such as enhanced acetylation of H3K9 at promoters of genes involved in interferon regulatory factors, inflammation, apoptosis, and oxidative stresses occurring in monocytes. Also, DNA methylation changes have been linked with diabetic vascular complications such as hypermethylation in the UNC13B promoter present in patients affected by diabetic nephropathy. DNA methylation has been linked to glycemic memory and diabetic retinopathy [160]. Indeed, the promoter of POLG1 was shown to be hypermethylated and associated to glycemic memory [161]. Inflammation is a typical pathological status due to a response to pathogen attacks and tissue offense. This process is modulated by different environmental factors such as enhanced expression of Toll-like receptors (TLRs) and upregulation of epigenetic modifications of RAGE [162]. Viral meningoencephalitis induced an inflammatory status that is associated with the induction of methyl-CpG protein 2 (MeCP2), an epigenetic factor essential for methylation of DNA. In the early stage of this disease, Purkinje cells have receptors recognized by pathogens that induce self-destructive mechanisms in these neurons. In later stages of viral meningoencephalitis, RAGE protein was observed in the adult brain and aging patients implying that the inflammatory process might be modulated by numerous posttranslationally modified proteins moving to the brain after binding with activated RAGE [163]. The effect of inflammation is the elimination of necrotic cells and the induction of repair pathways. However, the activation of proinflammatory proteins may provoke chronic diseases. Endogenous proteins may deregulate inflammation binding to RAGE and move to inflamed tissues where they regulate defense immune responses. Inflammation may be modulated by epigenetic modifications such as the activity of DNA methyltransferases such as methyl-CpG binding proteins (MeCP2), histone-modifying enzymes, and chromatin remodeling proteins and protein complexes. The most associated DNA methylation change is occurring at cytosine that affects gene expression and genomic imprinting [164] linked to cancer and mental retardation disorders.

Diabetic retinopathy (DR) is a pathological status that is caused by an enhanced incidence of diabetes mellitus. Therapeutic treatments are scarce [165]. Studies have shown that DR may be due to persistent epigenetic modifications because the improvement of diabetes mellitus does not imply beneficial effects on DR even in the long term. This is corroborated by other evidence that has linked epigenetic modifications to complications of diabetes [166].

Thioredoxin-interacting protein (TXNIP) has been linked to the promotion of diabetes and its vascular pathological effects. The binding of S100B and the RAGE promote the expression of TXNIP and inflammatory genes such as Cox2, VEGF-A, and ICAM-1. TXNIP-induced inflammation has been linked to nine histone modifications on H3 lysine. The p38 MAPK-NF-*κ*B signaling pathway has been involved in inflammation driven by TXNIP. Induction of TXNIP in endothelial cells promotes inflammation, silences H3K9 trimethylation, and enhances H3K9 acetylation at the proximal promoter of Cox2 [167].

In conclusion, in this review, we have briefly described the current methodologies for AGE's detection highlighting advantages and disadvantages in addition to essential principles in AGE's biochemistry, signaling, and epigenetic effects. Efforts in AGE's identification using these techniques to discover reliable aging AGR-related markers are ongoing. These methodologies can be divided into two main categories: chemical and molecular biology. Aspartic acid racemization is probably the most accurate technique available [168], although epigenetic methods are also getting more popularity. The development of portable instruments for AGE's detection for an easy and rapid detection in blood or saliva will be highly desirable and might be soon introduced in the market. These systems might be based on protein, mRNA, or noncoding RNA (miRNA, lncRNA, etc.) detection once reliable markers will be discovered. These systems should be based on the analysis of a pool of biomarkers since one specific marker linked with AGE's increase will be hardly discovered.

## Figures and Tables

**Table 1:** Methods for measuring AGEs in the human samples. The advantages and disadvantages of various assessment methods.

Methods	Marker	Compartment	Advantages	Disadvantages
Fluorimetric method: *λ*_ex_ = 370 nm, *λ*_em_ = 440 nm	Fluorescent AGEs (pentosidine)	Serum, urine, saliva	(i) Simple (ii) Rapid method	(i) No detection of nonfluorescent AGEs (ii) Interference of non-AGE fluorophores (iii) Results expressed in arbitrary units
Autofluorescence spectroscopy: *λ*_ex_ = 370 nm, *λ*_em_ = 440 nm	Fluorescent AGEs (pentosidine) and other fluorescent AGEs	*In vivo* skin	(i) Noninvasive, simple, rapid method (ii) Applicable to clinical or epidemiological studies (iii) Significant correlation with AGEs measured by HPLC	(i) Major contribution in fluorescence comes from fluorescent AGEs (ii) AGE level is lower in dark skin than in fair skin (on equal condition)
HPLC	AGEs, pentosidine, CML, CEL, MG	Plasma, tissue	(i) A bit invasive (ii) Suitable for AGE monitoring	(i) More costly in time and efforts (ii) Only applicable to AGE with known biochemical structures
Gas chromatography coupled with mass spectrometry (GC-MS)	CML, CEL, etc.	Urine	(i) Sophisticated technique (ii) High sensitivity (iii) Provide valid and accurate results	(i) More expensive (ii) Trained personnel
LC-MS/MS	Nonvolatile compounds (e.g., CML, CEL, and MG)	Plasma, urine	(i) No derivatization step is required (ii) Sophisticated technique (iii) High sensitivity (iv) Provide valid and accurate results	(i) More expensive (ii) Trained personnel
UHPLC	AGEs, pentosidine, CML, CEL, MG	Plasma, tissue	(i) Rapid method (ii) Good resolution	(i) More expensive (ii) Trained personnel
ELISA	AGEs, pentosidine, CML, CEL, etc.	Serum, urine, tissue	(i) A bit invasive (ii) Simple, fast, inexpensive (iii) No needs for sophisticated laboratory equipment	(i) Lack of enough antibody specificity (ii) Interference with glycation-free adducts
Western blotting	Antibodies against different molecules	Any tissues	(i) Economic (ii) Highly specific	(i) Complex procedure (ii) Low quantitative (iii) Accuracy

## References

[1] Semba R. D., Nicklett E. J., Ferrucci L. (2010). Does accumulation of advanced glycation end products contribute to the aging phenotype?. *The Journals of Gerontology Series A: Biological Sciences and Medical Sciences*.

[2] Uribarri J., Cai W., Peppa M. (2007). Circulating glycotoxins and dietary advanced glycation endproducts: two links to inflammatory response, oxidative stress, and aging. *The Journals of Gerontology Series A: Biological Sciences and Medical Sciences*.

[3] Ottum M. S., Mistry A. M. (2015). Advanced glycation end-products: modifiable environmental factors profoundly mediate insulin resistance. *Journal of Clinical Biochemistry and Nutrition*.

[4] Luevano-Contreras C., Chapman-Novakofski K. (2010). Dietary advanced glycation end products and aging. *Nutrients*.

[5] Poulsen M. W., Hedegaard R. V., Andersen J. M. (2013). Advanced glycation endproducts in food and their effects on health. *Food and Chemical Toxicology*.

[6] Vlassara H., Uribarri J., Cai W., Striker G. (2008). Advanced glycation end product Homeostasis. *Annals of the New York Academy of Sciences*.

[7] Del Turco S., Basta G. (2012). An update on advanced glycation endproducts and atherosclerosis. *BioFactors*.

[8] Ahmad S., Khan H., Siddiqui Z. (2018). AGEs, RAGEs and s-RAGE; friend or foe for cancer. *Seminars in Cancer Biology*.

[9] Walker D., Lue L. R., Paul G., Patel A., Sabbagh M. N. (2015). Receptor for advanced glycation endproduct modulators: a new therapeutic target in Alzheimer’s disease. *Expert Opinion on Investigational Drugs*.

[10] Takeuchi M., Yamagishi S. (2009). Involvement of toxic AGEs (TAGE) in the pathogenesis of diabetic vascular complications and Alzheimer’s disease. *Journal of Alzheimer's Disease*.

[11] Sorci G., Riuzzi F., Giambanco I., Donato R. (2013). RAGE in tissue homeostasis, repair and regeneration. *Biochimica et Biophysica Acta (BBA) - Molecular Cell Research*.

[12] Chavakis T., Bierhaus A., Nawroth P. (2004). RAGE (receptor for advanced glycation end products): a central player in the inflammatory response. *Microbes and Infection*.

[13] Asadipooya K., Uy E. M. (2019). Advanced glycation end products (AGEs), receptor for AGEs, diabetes, and bone: review of the literature. *Journal of the Endocrine Society*.

[14] Wautier M. P., Chappey O., Corda S., Stern D. M., Schmidt A. M., Wautier J. L. (2001). Activation of NADPH oxidase by AGE links oxidant stress to altered gene expression via RAGE. *American Journal of Physiology-Endocrinology and Metabolism*.

[15] Horiuchi S., Sakamoto Y., Sakai M. (2003). Scavenger receptors for oxidized and glycated proteins. *Amino Acids*.

[16] Cai W., He J. C., Zhu L., Chen X., Striker G. E., Vlassara H. (2008). AGE-receptor-1 counteracts cellular oxidant stress induced by AGEs via negative regulation of p66*^shc^*-dependent FKHRL1 phosphorylation. *American Journal of Physiology-Cell Physiology*.

[17] Kalea A. Z., Schmidt A. M., Hudson B. I. (2009). RAGE: a novel biological and genetic marker for vascular disease. *Clinical Science*.

[18] Smit A. J., Lutgers H. L. (2004). The clinical relevance of advanced glycation endproducts (AGE) and recent developments in pharmaceutics to reduce AGE accumulation. *Current Medicinal Chemistry*.

[19] Santilli F., D'Ardes D., Davì G. (2015). Oxidative stress in chronic vascular disease: from prediction to prevention. *Vascular Pharmacology*.

[20] Tessier F. J. (2010). The Maillard reaction in the human body. The main discoveries and factors that affect glycation. *Pathologie Biologie*.

[21] Ahmed N., Argirov O. K., Minhas H. S., Cordeiro C. A., Thornalley P. J. (2002). Assay of advanced glycation endproducts (AGEs): surveying AGEs by chromatographic assay with derivatization by 6-aminoquinolyl-N-hydroxysuccinimidyl-carbamate and application to N*ε*-carboxymethyl-lysine- and N*ε*-(1-carboxyethyl)lysine-modified albumin. *Biochemical Journal*.

[22] Nagai R., Fujiwara Y., Mera K., Yamagata K., Sakashita N., Takeya M. (2008). Immunochemical detection of N^*ε*^-(carboxyethyl)lysine using a specific antibody. *Journal of Immunological Methods*.

[23] Kaur S., Zilmer K., Leping V., Zilmer M. (2013). Serum methylglyoxal level and its association with oxidative stress and disease severity in patients with psoriasis. *Archives of Dermatological Research*.

[24] Uribarri J., Peppa M., Cai W. (2003). Restriction of dietary glycotoxins reduces excessive advanced glycation end products in renal failure patients. *Journal of the American Society of Nephrology*.

[25] Meerwaldt R., Graaff R., Oomen P. H. N. (2004). Simple non-invasive assessment of advanced glycation endproduct accumulation. *Diabetologia*.

[26] Wendel U., Persson N., Risinger C. (2018). A novel monoclonal antibody targeting carboxymethyllysine, an advanced glycation end product in atherosclerosis and pancreatic cancer. *PLoS One*.

[27] Finco A. B., Machado-de-Ávila R. A., Maciel R. (2016). Generation and characterization of monoclonal antibody against advanced glycation end products in chronic kidney disease. *Biochemistry and Biophysics Reports*.

[28] Baynes J. W. (2003). Chemical modification of proteins by lipids in diabetes. *Clinical Chemistry and Laboratory Medicine*.

[29] Maasen K., van Greevenbroek M. M. J., Scheijen J. L. J. M., van der Kallen C. J. H., Stehouwer C. D. A., Schalkwijk C. G. (2019). High dietary glycemic load is associated with higher concentrations of urinary advanced glycation endproducts: the Cohort on Diabetes and Atherosclerosis Maastricht (CODAM) Study. *The American Journal of Clinical Nutrition*.

[30] Vistoli G., De Maddis D., Cipak A., Zarkovic N., Carini M., Aldini G. (2013). Advanced glycoxidation and lipoxidation end products (AGEs and ALEs): an overview of their mechanisms of formation. *Free Radical Research*.

[31] Martin-Ventura J., Rodrigues-Diez R., Martinez-Lopez D., Salaices M., Blanco-Colio L., Briones A. (2017). Oxidative stress in Human Atherothrombosis: Sources, Markers and therapeutic targets. *International Journal of Molecular Sciences*.

[32] Corica D., Aversa T., Ruggeri R. M. (2019). Could AGE/RAGE-related oxidative homeostasis dysregulation enhance susceptibility to pathogenesis of cardio-metabolic complications in childhood obesity?. *Frontiers in Endocrinology*.

[33] Brás I. C., König A., Outeiro T. F. (2019). Glycation in Huntington’s disease: a possible modifier and target for intervention. *Journal of Huntington's Disease*.

[34] Li J., Liu D., Sun L., Lu Y., Zhang Z. (2012). Advanced glycation end products and neurodegenerative diseases: mechanisms and perspective. *Journal of the Neurological Sciences*.

[35] Heidari F., Rabizadeh S., Mansournia M. A. (2019). Inflammatory, oxidative stress and anti-oxidative markers in patients with endometrial carcinoma and diabetes. *Cytokine*.

[36] Walter K. R., Ford M. E., Gregoski M. J. (2019). Advanced glycation end products are elevated in estrogen receptor-positive breast cancer patients, alter response to therapy, and can be targeted by lifestyle intervention. *Breast Cancer Research and Treatment*.

[37] Dei R., Takeda A., Niwa H. (2002). Lipid peroxidation and advanced glycation end products in the brain in normal aging and in Alzheimer’s disease. *Acta Neuropathologica*.

[38] Cordain L., Eaton S. B., Sebastian A. (2005). Origins and evolution of the western diet: health implications for the 21st century. *The American Journal of Clinical Nutrition*.

[39] Nursten H. E. (2005). *The Maillard Reaction. Chemistry, Biochemistry, and Implications*.

[40] Thorpe S. R., Baynes J. W. (2003). Maillard reaction products in tissue proteins: new products and new perspectives. *Amino Acids*.

[41] Fukami K., Yamagishi S. I., Okuda S. (2014). Role of AGEs-RAGE system in cardiovascular disease. *Current Pharmaceutical Design*.

[42] Sell D. R., Monnier V. M. (1989). Isolation, purification and partial characterization of novel fluorophores from aging human insoluble collagen-rich tissue. *Connective Tissue Research*.

[43] Suliman M. E., Heimbürger O., Bárány P. (2003). Plasma pentosidine is associated with inflammation and malnutrition in end-stage renal disease patients starting on dialysis therapy. *Journal of the American Society of Nephrology*.

[44] van Deemter M., Ponsioen T. L., Bank R. A. (2009). Pentosidine accumulates in the aging vitreous body: a gender effect. *Experimental Eye Research*.

[45] Miller A. G., Meade S. J., Gerrard J. A. (2003). New insights into protein crosslinking via the Maillard reaction: structural requirements, the effect on enzyme function, and predicted efficacy of crosslinking inhibitors as anti-ageing therapeutics. *Bioorganic & Medicinal Chemistry*.

[46] Klaus A., Rau R., Glomb M. A. (2018). Modification and cross-linking of proteins by glycolaldehyde and glyoxal: a model system. *Journal of Agricultural and Food Chemistry*.

[47] Nasiri R., Field M. J., Zahedi M., Moosavi-Movahedi A. A. (2011). Cross-linking mechanisms of arginine and lysine with *α*,*β*-dicarbonyl compounds in aqueous solution. *The Journal of Physical Chemistry. A*.

[48] Gautieri A., Passini F. S., Silván U. (2017). Advanced glycation end-products: mechanics of aged collagen from molecule to tissue. *Matrix Biology*.

[49] Gugliucci A. (2017). Formation of fructose-mediated advanced glycation end products and their roles in metabolic and inflammatory diseases. *Advances in Nutrition: An International Review Journal*.

[50] Karlík M., Valkovič P., Hančinová V., Krížová L., Tóthová Ľ., Celec P. (2015). Markers of oxidative stress in plasma and saliva in patients with multiple sclerosis. *Clinical Biochemistry*.

[51] Raposeiras-Roubín S., Rodino-Janeiro B. K., Paradela-Dobarro B. (2015). Advanced glycation end-products as long-term predictors of death and reinfarction after an acute coronary syndrome. *Biomarkers in Medicine*.

[52] Thomas C. J., Cleland T. P., Sroga G. E., Vashishth D. (2018). Accumulation of carboxymethyl-lysine (CML) in human cortical bone. *Bone*.

[53] Liman P. B., Agustina R., Djuwita R. (2019). Dietary and plasma carboxymethyl lysine and tumor necrosis factor-*α* as mediators of body mass index and waist circumference among women in Indonesia. *Nutrients*.

[54] Vlassara H., Uribarri J. (2014). Advanced glycation end products (AGE) and diabetes: cause, effect, or both?. *Current Diabetes Reports*.

[55] Hull G. L. J., Woodside J. V., Ames J. M., Cuskelly G. J. (2012). N^*ε*^-(carboxymethyl)lysine content of foods commonly consumed in a Western style diet. *Food Chemistry*.

[56] Henning C., Glomb M. A. (2016). Pathways of the Maillard reaction under physiological conditions. *Glycoconjugate Journal*.

[57] Bierhaus A., Humpert P. M., Morcos M. (2005). Understanding RAGE, the receptor for advanced glycation end products. *Journal of Molecular Medicine*.

[58] Nemet I., Varga-Defterdarović L., Turk Z. (2006). Methylglyoxal in food and living organisms. *Molecular Nutrition & Food Research*.

[59] Elosta A., Ghous T., Ahmed N. (2012). Natural products as anti-glycation agents: possible therapeutic potential for diabetic complications. *Current Diabetes Reviews*.

[60] Temma J., Matsuhisa M., Horie T. (2015). Non-invasive measurement of skin autofluorescence as a beneficial surrogate marker for atherosclerosis in patients with type 2 diabetes. *The Journal of Medical Investigation*.

[61] Li X., Zheng T., Sang S., Lv L. (2014). Quercetin inhibits advanced glycation end product formation by trapping methylglyoxal and glyoxal. *Journal of Agricultural and Food Chemistry*.

[62] Tahara N., Yamagishi S., Takeuchi M. (2012). Positive association between serum level of glyceraldehyde-derived advanced glycation end products and vascular inflammation evaluated by [^18^F]Fluorodeoxyglucose positron emission tomography. *Diabetes Care*.

[63] Kajikawa M., Nakashima A., Fujimura N. (2015). Ratio of serum levels of AGEs to soluble form of RAGE is a predictor of endothelial function. *Diabetes Care*.

[64] Ueda S., Yamagishi S., Matsui T. (2012). Serum levels of advanced glycation end products (AGEs) are inversely associated with the number and migratory activity of circulating endothelial progenitor cells in apparently healthy subjects. *Cardiovascular Therapeutics*.

[65] Choei H., Sasaki N., Takeuchi M. (2004). Glyceraldehyde-derived advanced glycation end products in Alzheimer’s disease. *Acta Neuropathologica*.

[66] Matsui T., Oda E., Higashimoto Y., Yamagishi S. (2015). Glyceraldehyde-derived pyridinium (GLAP) evokes oxidative stress and inflammatory and thrombogenic reactions in endothelial cells via the interaction with RAGE. *Cardiovascular Diabetology*.

[67] Yamagishi S.-i., Nakamura N., Suematsu M., Kaseda K., Matsui T. (2015). Advanced glycation end products: a molecular target for vascular complications in diabetes. *Molecular Medicine*.

[68] Kong S. Y., Takeuchi M., Hyogo H. (2015). The association between glyceraldehyde-derived advanced glycation end-products and colorectal cancer risk. *Cancer Epidemiology Biomarkers & Prevention*.

[69] López-Díez R., Shekhtman A., Ramasamy R., Schmidt A. M. (2016). Cellular mechanisms and consequences of glycation in atherosclerosis and obesity. *Biochimica et Biophysica Acta (BBA) - Molecular Basis of Disease*.

[70] Xue M., Rabbani N., Thornalley P. J. (2011). Glyoxalase in ageing. *Seminars in Cell & Developmental Biology*.

[71] da-Cunha M. V., Jacquemin P., Delpierre G. (2006). Increased protein glycation in fructosamine 3-kinase-deficient mice. *Biochemical Journal*.

[72] Sessa L., Gatti E., Zeni F. (2014). The receptor for advanced glycation end-products (RAGE) is only present in mammals, and belongs to a family of cell adhesion molecules (CAMs). *PLoS One*.

[73] Carlo-Stella N., Bozzini S., De Silvestri A. (2009). Molecular study of receptor for advanced glycation endproduct gene promoter and identification of specific HLA haplotypes possibly involved in chronic fatigue syndrome. *International Journal of Immunopathology and Pharmacology*.

[74] Prasad C., Davis K. E., Imrhan V., Juma S., Vijayagopal P. (2019). Advanced glycation end products and risks for chronic diseases: intervening through lifestyle modification. *American Journal of Lifestyle Medicine*.

[75] Dahlmann M., Okhrimenko A., Marcinkowski P. (2014). RAGE mediates S100A4-induced cell motility via MAPK/ERK and hypoxia signaling and is a prognostic biomarker for human colorectal cancer metastasis. *Oncotarget*.

[76] Ando K., Sakoda M., Ueno S. (2018). Clinical implication of the relationship between high mobility group box-1 and tumor differentiation in hepatocellular carcinoma. *Anticancer Research*.

[77] Cai Z., Liu N., Wang C. (2016). Role of RAGE in Alzheimer’s disease. *Cellular and Molecular Neurobiology*.

[78] Prasad K., Mishra M. (2018). AGE–RAGE stress, stressors, and antistressors in health and disease. *International Journal of Angiology*.

[79] Daffu G., del Pozo C., O'Shea K., Ananthakrishnan R., Ramasamy R., Schmidt A. (2013). Radical roles for RAGE in the pathogenesis of oxidative stress in cardiovascular diseases and beyond. *International Journal of Molecular Sciences*.

[80] Younessi P., Yoonessi A. (2011). Advanced glycation end-products and their receptor-mediated roles: inflammation and oxidative stress. *Iranian Journal of Medical Sciences*.

[81] Uribarri J., Woodruff S., Goodman S. (2010). Advanced glycation end products in foods and a practical guide to their reduction in the diet. *Journal of the American Dietetic Association*.

[82] Chen J.-H., Lin X., Bu C., Zhang X. (2018). Role of advanced glycation end products in mobility and considerations in possible dietary and nutritional intervention strategies. *Nutrition & Metabolism*.

[83] Shangari N., O’Brien P. J. (2004). The cytotoxic mechanism of glyoxal involves oxidative stress. *Biochemical Pharmacology*.

[84] He C. J., Koschinsky T., Buenting C., Vlassara H. (2001). Presence of diabetic complications in type 1 diabetic patients correlates with low expression of mononuclear cell AGE-receptor-1 and elevated serum AGE. *Molecular Medicine*.

[85] Chang J. B., Chu N. F., Syu J. T., Hsieh A. T., Hung Y. R. (2011). Advanced glycation end products (AGEs) in relation to atherosclerotic lipid profiles in middle-aged and elderly diabetic patients. *Lipids in Health and Disease*.

[86] Cai W., He J. C., Zhu L. (2004). High levels of dietary advanced glycation end products transform low-density lipoprotein into a potent redox-sensitive mitogen-activated protein kinase stimulant in diabetic patients. *Circulation*.

[87] Lopes-Virella M. F., Baker N. L., Hunt K. J. (2013). Baseline markers of inflammation are associated with progression to macroalbuminuria in type 1 diabetic subjects. *Diabetes Care*.

[88] Peng Z., Yang X., Qin J. (2017). Glyoxalase-1 overexpression reverses defective proangiogenic function of diabetic adipose-derived stem cells in streptozotocin-induced diabetic mice model of critical limb ischemia. *Stem Cells Translational Medicine*.

[89] Chawla D., Bansal S., Banerjee B. D., Madhu S. V., Kalra O. P., Tripathi A. K. (2014). Role of advanced glycation end product (AGE)-induced receptor (RAGE) expression in diabetic vascular complications. *Microvascular Research*.

[90] Chuah Y. K., Basir R., Talib H., Tie T. H., Nordin N. (2013). Receptor for advanced glycation end products and its involvement in inflammatory diseases. *International Journal of Inflammation*.

[91] Ott C., Jacobs K., Haucke E., Navarrete Santos A., Grune T., Simm A. (2014). Role of advanced glycation end products in cellular signaling. *Redox Biology*.

[92] Gebhardt C., Riehl A., Durchdewald M. (2008). RAGE signaling sustains inflammation and promotes tumor development. *Journal of Experimental Medicine*.

[93] Sparvero L. J., Asafu-Adjei D., Kang R. (2009). RAGE (receptor for advanced glycation endproducts), RAGE ligands, and their role in cancer and inflammation. *Journal of Translational Medicine*.

[94] Korwar A. M., Bhonsle H. S., Chougale A. D. (2012). Analysis of AGE modified proteins and RAGE expression in HER2/neu negative invasive ductal carcinoma. *Biochemical and Biophysical Research Communications*.

[95] Sanajou D., Ghorbani Haghjo A., Argani H., Aslani S. (2018). AGE-RAGE axis blockade in diabetic nephropathy: current status and future directions. *European Journal of Pharmacology*.

[96] Miyazaki A., Nakayama H., Horiuchi S. (2002). Scavenger receptors that recognize advanced glycation end products. *Trends in Cardiovascular Medicine*.

[97] Tam X. H. L., Shiu S. W. M., Leng L., Bucala R., Betteridge D. J., Tan K. C. B. (2011). Enhanced expression of receptor for advanced glycation end-products is associated with low circulating soluble isoforms of the receptor in type 2 diabetes. *Clinical Science*.

[98] Ivancovsky-Wajcman D., Zelber-Sagi S., Isakov N. F. (2019). Serum soluble receptor for AGE (sRAGE) levels are associated with unhealthy lifestyle and nonalcoholic fatty liver disease. *Clinical and Translational Gastroenterology*.

[99] Wannamethee S. G., Welsh P., Papacosta O. (2017). Circulating soluble receptor for advanced glycation end product: Cross-sectional associations with cardiac markers and subclinical vascular disease in older men with and without diabetes. *Atherosclerosis*.

[100] Bucciarelli L. G., Wendt T., Qu W. (2002). RAGE blockade stabilizes established atherosclerosis in diabetic apolipoprotein E–null mice. *Circulation*.

[101] Barile G. R., Pachydaki S. I., Tari S. R. (2005). The RAGE axis in early diabetic retinopathy. *Investigative Ophthalmology & Visual Science*.

[102] Yamagishi S., Matsui T. (2010). Soluble form of a receptor for advanced glycation end products (sRAGE) as a biomarker. *Frontiers in Bioscience*.

[103] Falcone C., Bozzini S., Guasti L. (2013). Soluble RAGE plasma levels in patients with coronary artery disease and peripheral artery disease. *The Scientific World Journal*.

[104] Gopal P., Rutten E. P. A., Dentener M. A., Wouters E. F. M., Reynaert N. L. (2012). Decreased plasma sRAGE levels in COPD: influence of oxygen therapy. *European Journal of Clinical Investigation*.

[105] Steenvoorden M. M. C., Huizinga T. W. J., Verzijl N. (2006). Activation of receptor for advanced glycation end products in osteoarthritis leads to increased stimulation of chondrocytes and synoviocytes. *Arthritis & Rheumatism*.

[106] Hung J. H., Su I. J., Lei H. Y. (2004). Endoplasmic reticulum stress stimulates the expression of cyclooxygenase-2 through activation of NF-*κ*B and pp38 mitogen-activated protein kinase. *Journal of Biological Chemistry*.

[107] Rasheed Z., Akhtar N., Haqqi T. M. (2011). Advanced glycation end products induce the expression of interleukin-6 and interleukin-8 by receptor for advanced glycation end product-mediated activation of mitogen-activated protein kinases and nuclear factor-*κ*B in human osteoarthritis chondrocytes. *Rheumatology*.

[108] Jiang H. Y., Wek S. A., McGrath B. C. (2003). Phosphorylation of the alpha subunit of eukaryotic initiation factor 2 is required for activation of NF-*κ*B in response to diverse cellular stresses. *Molecular and Cellular Biology*.

[109] Waris G., Tardif K. D., Siddiqui A. (2002). Endoplasmic reticulum (ER) stress: hepatitis C virus induces an ER-nucleus signal transduction pathway and activates NF-*κ*B and STAT-3. *Biochemical Pharmacology*.

[110] Yang L., Carlson S. G., McBurney D., Horton W. E. (2005). Multiple signals induce endoplasmic reticulum stress in both primary and immortalized chondrocytes resulting in loss of differentiation, impaired cell growth, and apoptosis. *Journal of Biological Chemistry*.

[111] Liang S. H., Zhang W., McGrath B. C., Zhang P., Cavener D. R. (2006). PERK (eIF2*α* kinase) is required to activate the stress-activated MAPKs and induce the expression of immediate-early genes upon disruption of ER calcium homoeostasis. *Biochemical Journal*.

[112] Zhang Y., Huang X., Yuan Y. (2019). Linagliptin protects human chondrogenic ATDC5 cells against advanced glycation end products (AGEs)-induced apoptosis via a mitochondria-dependent pathway. *Chemico-Biological Interactions*.

[113] Mei Y. M., Li L., Wang X. Q. (2019). AGEs induces apoptosis and autophagy via reactive oxygen species in human periodontal ligament cells. *Journal of Cellular Biochemistry*.

[114] Nakamura K., Yamagishi S., Adachi H. (2007). Serum levels of sRAGE, the soluble form of receptor for advanced glycation end products, are associated with inflammatory markers in patients with type 2 diabetes. *Molecular Medicine*.

[115] Prasad K. (2014). Low levels of serum soluble receptors for advanced glycation end products, biomarkers for disease state: myth or reality. *International Journal of Angiology*.

[116] Koyama H., Shoji T., Yokoyama H. (2005). Plasma level of endogenous secretory RAGE is associated with components of the metabolic syndrome and atherosclerosis. *Arteriosclerosis, Thrombosis, and Vascular Biology*.

[117] Münch G., Keis R., Wessels A. (1997). Determination of advanced glycation end products in serum by fluorescence spectroscopy and competitive ELISA. *Clinical Chemistry and Laboratory Medicine*.

[118] Scheijen J. L. J. M., Clevers E., Engelen L. (2016). Analysis of advanced glycation endproducts in selected food items by ultra-performance liquid chromatography tandem mass spectrometry: presentation of a dietary AGE database. *Food Chemistry*.

[119] Badoud R., Fay L., Richli U., Hušek P. (1991). Gas chromatographic determination of N-carboxymethyl amino acids, the periodate oxidation products of Amadori compounds. *Journal of Chromatography A*.

[120] Shoji N., Nakagawa K., Asai A. (2010). LC-MS/MS analysis of carboxymethylated and carboxyethylated phosphatidylethanolamines in human erythrocytes and blood plasma. *Journal of Lipid Research*.

[121] Lee J. S., Chung Y. S., Chang S. Y., Jung Y. S., Kim S. H. (2017). Simple Quantification of Pentosidine in Human Urine and Plasma by High-Performance Liquid Chromatography. *International Journal of Analytical Chemistry*.

[122] Assar S. H., Moloney C., Lima M., Magee R., Ames J. M. (2009). Determination of N^*ε*^ (carboxymethyl)lysine in food systems by ultra performance liquid chromatography-mass spectrometry. *Amino Acids*.

[123] Taneda S., Monnier V. M. (1994). ELISA of pentosidine, an advanced glycation end product, in biological specimens. *Clinical Chemistry*.

[124] Matsui T., Joo H. D., Lee J. M. (2015). Development of a monoclonal antibody-based ELISA system for glyceraldehyde-derived advanced glycation end products. *Immunology Letters*.

[125] Röcken C., Kientsch-Engel R., Mansfeld S. (2003). Advanced glycation end products and receptor for advanced glycation end products in AA amyloidosis. *The American Journal of Pathology*.

[126] Koetsier M., Nur E., Chunmao H. (2010). Skin color independent assessment of aging using skin autofluorescence. *Optics Express*.

[127] Meerwaldt R., Lutgers H., Links T. (2007). Skin autofluorescence is a strong predictor of cardiac mortality in diabetes. *Diabetes Care*.

[128] Gerrits E. G., Lutgers H. L., Kleefstra N. (2008). Skin autofluorescence: a tool to identify type 2 diabetic patients at risk for developing microvascular complications. *Diabetes Care*.

[129] Mulder D. J., Water T. V., Lutgers H. L. (2006). Skin autofluorescence, a novel marker for glycemic and oxidative stress-derived advanced glycation endproducts: an overview of current clinical studies, evidence, and limitations. *Diabetes Technology & Therapeutics*.

[130] Smit A. J., Gerrits E. G. (2010). Skin autofluorescence as a measure of advanced glycation endproduct deposition: a novel risk marker in chronic kidney disease. *Current Opinion in Nephrology and Hypertension*.

[131] Nowotny K., Jung T., Höhn A., Weber D., Grune T. (2015). Advanced glycation end products and oxidative stress in type 2 diabetes mellitus. *Biomolecules*.

[132] Jeong B., Jung C. H., Lee Y. H. (2016). A novel imaging platform for non-invasive screening of abnormal glucose tolerance. *Diabetes Research and Clinical Practice*.

[133] Menini S., Iacobini C., de Latouliere L. (2018). The advanced glycation end-product N^*ε*^-carboxymethyllysine promotes progression of pancreatic cancer: implications for diabetes-associated risk and its prevention. *The Journal of Pathology*.

[134] Agalou S., Ahmed N., Babaei-Jadidi R., Dawnay A., Thornalley P. J. (2005). Profound mishandling of protein glycation degradation products in uremia and dialysis. *Journal of the American Society of Nephrology*.

[135] Yuan Y., Sun H., Sun Z. (2017). Advanced glycation end products (AGEs) increase renal lipid accumulation: a pathogenic factor of diabetic nephropathy (DN). *Lipids in Health and Disease*.

[136] Nagai R., Shirakawa J., Ohno R. (2016). Antibody-based detection of advanced glycation end-products: promises vs. limitations. *Glycoconjugate Journal*.

[137] Nagai R., Horiuchi S. (2003). Application of monoclonal antibody libraries for the measurement of glycation adducts. *Biochemical Society Transactions*.

[138] Rodríguez J. M., Leiva Balich L., Concha M. J. (2015). Reduction of serum advanced glycation end-products with a low calorie Mediterranean diet. *Nutrición Hospitalaria*.

[139] Takeuchi M., Takino J., Furuno S. (2015). Assessment of the concentrations of various advanced glycation end-products in beverages and foods that are commonly consumed in Japan. *PLoS One*.

[140] Koito W., Araki T., Horiuchi S., Nagai R. (2004). Conventional antibody against N^*ε*^-(carboxymethyl)lysine (CML) shows cross-reaction to N^*ε*^-(carboxyethyl)lysine (CEL): immunochemical quantification of CML with a specific antibody. *Journal of Biochemistry*.

[141] Thornalley P. J. (2005). Measurement of protein glycation, glycated peptides, and glycation free adducts. *Peritoneal Dialysis International*.

[142] Miki Hayashi C., Nagai R., Miyazaki K. (2002). Conversion of Amadori Products of the Maillard Reaction to N^*ε*^-(carboxymethyl)lysine by Short-Term Heating: Possible Detection of Artifacts by Immunohistochemistry. *Laboratory Investigation*.

[143] Perkins R., Miranda E., Karstoft K., Beisswenger P., Solomon T., Haus J. (2019). Experimental hyperglycemia alters circulating concentrations and renal clearance of oxidative and advanced glycation end products in healthy obese humans. *Nutrients*.

[144] Gómez-Ojeda A., Jaramillo-Ortíz S., Wrobel K. (2018). Comparative evaluation of three different ELISA assays and HPLC-ESI-ITMS/MS for the analysis of N^*ε*^-carboxymethyl lysine in food samples. *Food Chemistry*.

[145] Bass J. J., Wilkinson D. J., Rankin D. (2017). An overview of technical considerations for Western blotting applications to physiological research. *Scandinavian Journal of Medicine & Science in Sports*.

[146] Mishra M., Tiwari S., Gomes A. V. (2017). Protein purification and analysis: next generation Western blotting techniques. *Expert Review of Proteomics*.

[147] Ravelojaona V., Péterszegi G., Molinari J., Gesztesi J. L., Robert L. (2007). Démonstration de l'effet cytotoxique des produits avancés de la glycation (AGE-s). *Journal de la Société de Biologie*.

[148] Fetita L.-S., Sobngwi E., Serradas P., Calvo F., Gautier J.-F. (2006). Consequences of fetal exposure to maternal diabetes in offspring. *The Journal of Clinical Endocrinology & Metabolism*.

[149] El-Osta A., Brasacchio D., Yao D. (2008). Transient high glucose causes persistent epigenetic changes and altered gene expression during subsequent normoglycemia. *The Journal of Experimental Medicine*.

[150] Zhou L., Wang W., Yang C. (2018). GADD45a promotes active DNA demethylation of the MMP-9 promoter via base excision repair pathway in AGEs-treated keratinocytes and in diabetic male rat skin. *Endocrinology*.

[151] Piperi C. (2017). Dietary advanced glycation end-products: molecular mechanisms and preventive tools. *Current Nutrition Reports*.

[152] Brownlee M. (2005). The pathobiology of diabetic complications: a unifying mechanism. *Diabetes*.

[153] Brings S., Fleming T., Freichel M., Muckenthaler M. U., Herzig S., Nawroth P. P. (2017). Dicarbonyls and advanced glycation end-products in the development of diabetic complications and targets for intervention. *International Journal of Molecular Sciences*.

[154] Reddy M. A., Tak Park J., Natarajan R. (2013). Epigenetic modifications in the pathogenesis of diabetic nephropathy. *Seminars in Nephrology*.

[155] Nakatani Y., Inagi R. (2016). Epigenetic regulation through SIRT1 in podocytes. *Current Hypertension Reviews*.

[156] Holman R. R., Paul S. K., Bethel M. A., Matthews D. R., Neil H. A. W. (2008). 10-year follow-up of intensive glucose control in type 2 diabetes. *The New England Journal of Medicine*.

[157] Duckworth W., Abraira C., Moritz T. (2009). Glucose control and vascular complications in veterans with type 2 diabetes. *The New England Journal of Medicine*.

[158] Babizhayev M. A., Strokov I. A., Nosikov V. V. (2015). The role of oxidative stress in diabetic neuropathy: generation of free radical species in the glycation reaction and gene polymorphisms encoding antioxidant enzymes to genetic susceptibility to diabetic neuropathy in population of type I diabetic patients. *Cell Biochemistry and Biophysics*.

[159] Brasacchio D., Okabe J., Tikellis C. (2009). Hyperglycemia induces a dynamic cooperativity of histone methylase and demethylase enzymes associated with gene-activating epigenetic marks that coexist on the lysine tail. *Diabetes*.

[160] Al-Haddad R., Karnib N., Assaad R. A. (2016). Epigenetic changes in diabetes. *Neuroscience Letters*.

[161] Pradhan S., Chin H. G., Estève P. O., Jacobsen S. E. (2009). SET7/9 mediated methylation of non-histone proteins in mammalian cells. *Epigenetics*.

[162] Rajasekar P., O’Neill C. L., Eeles L., Stitt A. W., Medina R. J. (2015). Epigenetic changes in endothelial progenitors as a possible cellular basis for glycemic memory in diabetic vascular complications. *Journal Diabetes Research*.

[163] Tewari S., Zhong Q., Santos J. M., Kowluru R. A. (2012). Mitochondria DNA replication and DNA methylation in the metabolic memory associated with continued progression of diabetic retinopathy. *Investigative Ophthalmology & Visual Science*.

[164] Maslinska D., Laure-Kamionowska M., Maslinski S. (2014). Methyl-CpG binding protein 2, receptors of innate immunity and receptor for advanced glycation end-products in human viral meningoencephalitis. *Folia Neuropathologica*.

[165] Rodríguez-Dorantes M., Téllez-Ascencio N., Cerbón M. A., López M., Cervantes A. (2004). DNA methylation: an epigenetic process of medical importance. *Revista de Investigación Clínica*.

[166] Zeng J., Chen B. (2014). Epigenetic mechanisms in the pathogenesis of diabetic retinopathy. *Ophthalmologica*.

[167] Perrone L., Devi T. S., Hosoya K., Terasaki T., Singh L. P. (2009). Thioredoxin interacting protein (TXNIP) induces inflammation through chromatin modification in retinal capillary endothelial cells under diabetic conditions. *Journal of Cellular Physiology*.

[168] Zapico S., Stone R. (2019). The evolution of methodology in biochemical age estimation. *Age Estimation. Chapter 13. A Multidisciplinary Approach*.

